# Is the Glass Half Full or Half Empty? How to Reverse the Effect of Glass Elongation on the Volume Poured

**DOI:** 10.1371/journal.pone.0109374

**Published:** 2014-10-24

**Authors:** Simone R. Caljouw, Ruud van Wijck

**Affiliations:** Center for Human Movement Sciences, University Medical Center Groningen, University of Groningen, Groningen, the Netherlands; VU University Amsterdam, Netherlands

## Abstract

To reduce the volume of drinks and the risk of overconsumption, health professionals recommend the use of tall skinny instead of short wide glasses. Yet the results of the present study contradict this health advice. Participants who generously filled up a glass with lemonade served 9% more in tall narrow compared with short wide glasses (p<0.05). In addition, when pouring a small amount (i.e., a shot), participants poured 3% more in a short wide than in a tall narrow glass (p<0.05). Elongation may bias the perceived volume that is poured but also the perceived volume of the free space in the glass. We hypothesised that shifting attention from the bottom to the brim of the glass when filling it close to capacity might reverse the glass elongation effect on the quantity poured. This hypothesis was tested, by investigating two pouring tasks that differed in the required focus of attention. When the instruction was to match a reference volume, participants poured more liquid in the short wide compared with the tall narrow glass (p<0.05). The effect of glass elongation on poured volume was the opposite when the instruction was to leave space in the glasses for the reference volume. It seems likely that task and individual factors affect the pourer's viewing strategy and thus may determine the direction of the glass elongation effect on the volume poured.

## Introduction

It is far from obvious that visual illusions that invoke a perceptual bias should necessarily lead to a biased motor performance [1]. Yet there are many situations in which distance and size illusions can be used in environmental and object design to positively influence someone's performance. For example, birds arrange pebbles in front of their nests [2] and goal-keepers can mimic a Müller-Lyer illusion [3] to exaggerate their stature. Also in urban design, illusion configurations are used to slow traffic [4] or to prevent falls when climbing stairs [5]. Food marketers are well known for ‘fooling’ consumers to eat and buy more, but also in this industry there are opportunities to use visual illusions in more positive ways. An excess of caloric intake increases the risk of developing obesity and visual illusions can elegantly be used to reduce food intake without decreasing satisfaction [6]. There is ample evidence showing an association between the shape of tableware (e.g. plates, bowls, and other containers) and the amount of food people serve and eat [7].

Building on Piaget's experiments [8], Raghubir & Krishna [9] showed that people tend to focus on the height of a cylindrical object at the expense of its horizontal dimension when judging the volume of drinking glasses. Wansink and Van Ittersum [10], [11] linked this finding to consumption volumes, they observed that teenagers and adults poured more soda in short wide glasses than in tall narrow glasses with the same capacity. Given the concern of obesity, Wansink [12] wrote a bestseller (translated into 18 languages) that included the general advice to replace short wide glasses with tall narrow glasses to help reduce overconsumption (see also Oprah Winfrey [13]). Yet a pilot experiment from our lab in the Netherlands showed the opposite result: people poured more lemonade in a tall narrow glass than in a short wide glass [14]. Our observation was clearly inconsistent with the seminal work of the American researcher Wansink. Understanding the circumstances that can lead to a reversal in the glass elongation bias on the volume served will carry theoretical as well as managerial significance.

Recently it has been stated that an overreliance on a thin slice of humanity, that is well-educated subjects from the United States, can produce false claims about behavioral phenomena [15]. Already in the 60 s it was shown that adults from different cultural populations were differentially susceptible to illusions that bias length judgments, such as the Müller-Lyer illusion and the horizontal-vertical illusion [16]. This population level variability involved differences in the magnitude of the illusion bias and some populations could not even detect this illusion at all. We think that the reversed glass elongation bias on served volume observed in the Dutch sample is not related to a different developmental trajectory in the Dutch compared to the American society per se. Apart from cultural differences in the sample studied, research outcomes may also be influenced by cultural differences in the research environment, i.e. the instruments and tasks selected to assess a phenomenon may be culturally biased. A cultural difference that may have mitigated the glass shape effect on served volume may concern the size of the glasses used. Wansink and Van Ittersum [10] used extremely large American glasses of 22.3 fl oz (0.63 liters) whereas regular glasses in the Netherlands (and most other European countries) can contain only half the volume. Addressing this cultural difference in experimental design may provide key insights in the reversed bias of glass elongation on served volume.

**Figure 1:**
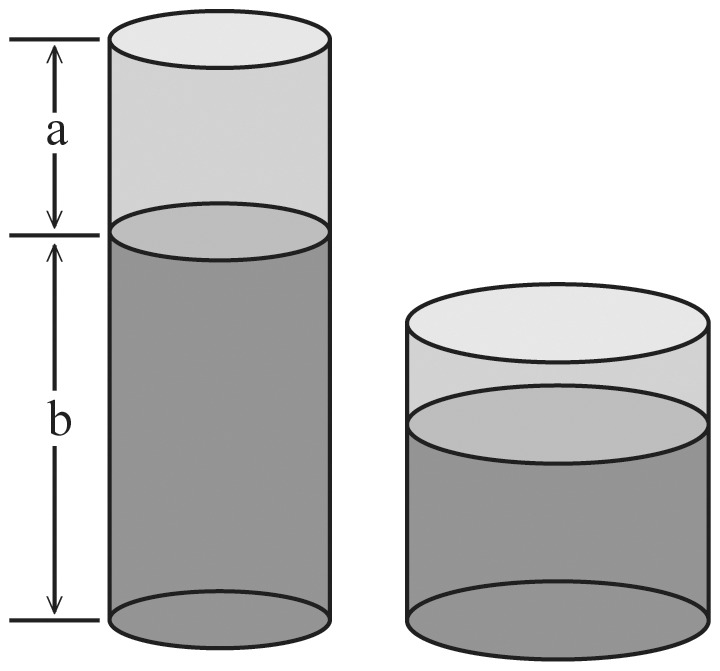
Two experimental glasses with the same capacity and amount of liquid (66% filled). Due to the effect of elongation both pouring volume (b) and rest volume (a) appear larger in the tall narrow glass than in the short wide glass.

Building on the notion that glass size mitigates the effect of glass elongation on served volume, we hypothesize that a reversed effect can be invoked by changing the portion size relative to the capacity of the glass (see Figure 1). When generously filling a glass, people might focus on the unfilled part of the glass to prevent the glass from overflowing. Elongation of this rest volume may positively bias the perceived capacity of the unfilled part of the glass and correspondingly less space may be spared in the tall skinny glass than in the short wide glass when filling. Accordingly, when using this *rest volume strategy* people serve more liquid in the tall narrow glass than in the short wide glass (i.e., a positive *glass* elongation effect). In contrast, when a glass is very large, people might focus on the poured volume to estimate whether they reached their intended volume. Exactly the same elongation bias may act on the filled part of the glass, positively influencing the perceived volume poured which prevents people from pouring more. So, when using this *intended volume strategy* less liquid is poured in the tall narrow glass than in the short wide glass (i.e. a negative *glass* elongation effect). To sum up, the same visual illusion of space elongation may influence the perceived capacity of the filled but also of the unfilled part of the glass. Changing the allocation of attention from the filled to the unfilled part of the glass when pouring a drink may reverse the glass elongation effect on the volume served. This triggers the question (1) whether and how pouring strategy differs between individuals and (2) how environmental constraints and instructions can impact pouring strategy and reverse the elongation effect.

The results of two experiments are presented. In the first experiment, participants helped themselves to some lemonade in a tall narrow and a short wide glass both with a regular capacity of 0.3 liters (10.1 fl oz). Participants were also asked to pour a smaller amount, equivalent to serving a shot, in the experimental glasses. A shadow experiment, with questionnaires on drinking and sports, was set-up to prevent participants from thinking about their pouring actions and the shape of the glasses. It was expected that glass elongation would differentially affect the volume served when filling a glass near or well below its capacity. In the second experiment, the *intended volume strategy* and the *rest volume strategy* were enforced by means of explicit instructions. Participants were required to fill or leave space for a designated amount in the two experimental glasses. A reversal of the glass elongation effect on the served volume was expected based on the strategy used.

## Experiment 1

The aim of experiment 1 was to assess the glass elongation effect on volume poured in a natural pouring task where glasses with a regular capacity of 0.3 liters (10.1 fl oz) would be filled sparingly and closer to capacity. In the context of a shadow experiment on drinking and sports (to create a natural task) students helped themselves to some lemonade in a tall narrow and a short wide glass. To assess whether participants would be differentially affected by the glass shape when pouring closer to capacity, participants were equally split in three groups based on the amount of drink they poured. It was expected that the frugal pourers, who serve more sparingly in relation to the capacity of the glass, would pour more in the short wide than the tall narrow glass and that the generous pourers, who fill closer to capacity, would show the reverse and pour more in the tall narrow than the short wide glass.

Prior research found a negative glass elongation bias when bartenders served an amount well below the capacity of the glass, as a mixed-drink in a tumbler (short wide glass) contained more alcohol than a mixed-drink in a highball (tall narrow) glass [11]. To verify this effect in our sample and task, participants were also asked to serve an amount of liquid in the experimental glasses that was equivalent to a shot of hard liquor.

### Methods

#### Participants

42 students (59.5% female, mean age 21.3 years, range 18–26) volunteered to participate in a study from the Center of Human Movement Sciences, University of Groningen about sports and drinking behaviour. Written informed consent was obtained from all participants involved in the study. The local ethics committee of the Center of Human Movement Sciences of the University Medical Center Groningen approved the experiment.

#### The glasses and instructions

Two glass shapes were evaluated in this study: a tall narrow glass (height 14.5 cm, unfilled weight 274 gr) and a short wide glass (height 7.5 cm, unfilled weight 315 gr). Both glasses could contain 0.3 liters (10.1 fl oz). Participants were required to pour lemonade from a 1.5 liters jug into a glass. First, they were instructed to pour themselves a glass of lemonade. Second, they were instructed to pour an amount of lemonade in the glass that equals a shot.

#### Set up and procedure

Participants took part in two, 15-minutes-long experimental sessions separated by 1 week. The two experimental sessions were identical with the exception that glasses of a different shape were presented and counterbalanced in order across participants. Participants were seated at a desk in a distraction free room. There was a questionnaire, a glass, and a 1.5L jug of lemonade on the table.

Participants were asked to answer questions with regard to their sports and drinking behaviour. They were asked to follow instructions on the questionnaire carefully and step by step. The “sport and drink” questionnaire consisted of questions and instructions. The first instruction was “serve yourself a glass of lemonade before answering any of the questions. Do not drink yet, the experimenter will weigh your filled glass”. In order to serve themselves a glass of lemonade participants picked up one of the two identical empty glasses in front of them and placed them in front of them to pour a drink. Haptic contact with the glasses was thus allowed. After helping themselves to some lemonade they answered questions and the experimenter weighed the glass. Participants assumed that the amount of lemonade they served was a reference measure for their daily beverage intake recall. Aids such as an actual amount of lemonade poured in a glass may help participants to more accurately estimate and report the amount of drink they consume. Part 2 of the questionnaire collected data on sporting and drinking behaviour. While answering these questions participants were allowed to drink the lemonade they served themselves. Part 3 of the survey started with the instruction “serve a shot in the other clean glass on your desk. A shot is an amount of liquid you normally would serve in a shot glass designed for spirits. Do not drink yet, the experimenter will weigh your filled glass”. In order to serve a shot of lemonade participants picked up the jug of lemonade and placed the other empty glass in front of them. After pouring a shot they answered questions and the experimenter weighed the glass. At the end of the session participants were asked to fill in a drinking diary during the next week, in which they had to note every drink. After a week they handed in their drinking diary and the same “sport and drink” survey was conducted, but this time 0.3 liters glasses of the other shape were presented. To test whether participants were aware of the purpose of the experiment, they were asked to explain what the experiment was about. None of the participants mentioned that different glasses of identical volume were used and that the aim was to study differences in the amount of lemonade they poured in the two different glasses. At the end we explained the rationale behind the experiment and asked their permission for reporting data on the pouring volume.

### Results

Based on the average pouring volume when serving a drink in both glass types, we classified the 42 participants in three equally sized groups: frugal pourers (n = 14), average pourers (n = 14), and generous pourers (n = 14). When serving a drink the 14 frugal pourers filled their glasses less than 66.1% (mean = 59%, sd = 6.5%) and the 14 generous pourers filled the glasses more than 71.5% (mean = 74.8%, sd = 2.7%), the other 14 participants were classified as average pourers (mean = 69%, sd = 1,4%). Table 1 shows for each glass shape (tall narrow and small wide) and instruction (“pour a drink” and “pour a shot”) the means and standard deviations of the volume served for each of the three groups. A RM-ANOVA was used to analyse the within-subjects effects of glass shape and instruction and the between-subjects effect of group. Post hoc paired t tests (to test the effect of instruction and glass shape) and ANOVA's (to test the effect of group) were performed.

**Table 1:** The mean quantity of lemonade poured and standard deviations (in grams), the mean percentage of the glass filled, the liquid level relative to the rim of the glass (in cm), the percentage of participants that poured more in the short wide than in the tall narrow glass (% pp_negative bias_) as a function of instruction (pour a drink versus a shot), type of pourer (generous versus frugal) and glass shape (tall narrow versus short wide).

Instruction	Pourer	Glass	Quantity (g)	% filled	Level (cm)	% pp_negative bias_
Drink	Frugal	tall	179.2 (27.6)	57	6.4	64
		short	192.5 (22.2)	61	5.1	
	Average	tall	228.9 (15.3)	73	4.1	29
		short	206.6 (19.9)	66	3.4	
	Generous	tall	249.8 (12.1)	79	3.1	7
		short	221.6 (14.1)	70	2.5	
Shot	Frugal	tall	36.5(15.0)	12	13.3	86
		short	41.5 (13.0)	13	6.6	
	Average	tall	44.6 (17.8)	14	12.7	64
		short	55.9 (28.3)	18	6.3	
	Generous	tall	31.6414.4)	10	13.1	64
		short	43.3 (16.6)	14	6.6	

#### Effect of Instruction

Overall, people served more when they were instructed to pour a drink than a shot as confirmed by the significant main effect of instruction (F(1,39) = 2989. 82, p<.001, η_p_^2^ = .99). Also the interaction of instruction×glass shape was significant (F(1,39) = 15.43, p<.001, η_p_^2^ = .28). When pouring a shot, more drink is poured in the short wide than in the tall narrow glass. In contrast, when asked to pour a drink, more lemonade is poured in the tall narrow glass than in the short wide glass.

#### Effect of group

Groups were classified in frugal, average and generous pourers based on the volume poured when serving a drink, hence a significant main effect of group on pouring volume was found (F(2,39) = 25.13, p<.001, η_p_^2^ = .56). Although pouring volume differed significantly between groups when pouring a drink (F(2,39) = 52.84, p<.001, η^2^ = .73), differences in pouring volume between groups when pouring a shot were small (F(2,39) = 2.69, p = .08, η^2^ = .12), resulting in a significant two-way interaction effect of group×instruction (F(2,39) = 20.22, p<.001, η_p_^2^ = .51).

#### Effect of glass type

The RM-ANOVA did not reveal a significant main effect of glass shape (F(1,39) = .41, p = .53, η_p_^2^ = .01). However, the interactions of glass shape×instruction (F(2,39) = 15.43, p<.001, η_p_^2^ = .28), glass shape×group (F(2,39) = 8.97, p = .001, η_p_^2^ = .32) and the three-way interaction of glass shape×instruction×group (F(2,39) = 4.47, p = .018, η_p_^2^ = .19) were all significant. When serving a shot all three groups (frugal, average and generous pourers) showed a *negative* bias of elongation on pouring volume. Less drink was served in the tall narrow than in the short wide glass. This *negative* glass elongation bias was significant for the average (t(13) = −2.15, p = .05, *d* = 0.57) and frugal pourers (t(13) = −2.46, p = .029, *d* = 0.65). When serving a drink only the frugal pourers showed a marginally significant tendency t(13) = −1.71, p = .10, *d* = 0.45) to a *negative* bias of glass elongation on served volume. In contrast, the generous (t(13) = 5.25, p<.001, d = 1.41) and average pourers (t(13) = 2.41, p = .03, d = 0.65) showed a *positive* bias of glass elongation on poured volume. When serving a larger quantity, more lemonade was poured in the tall narrow than in the short wide glass.

### Discussion

The primary result of Experiment 1 is that the glass elongation effect on served volume reverses depending upon filling the glass sparingly or generously. When participants served themselves a relatively large portion with respect to the capacity of the glass, participants poured significantly more in the tall narrow than in the short wide glass. In contrast, when participants served a relatively small portion (e.g., a shot), they poured more in the short wide than in the tall slender glass. These data question the health advice, delivered by popular media and based on prior research in the U.S, to prevent overconsumption of sugary beverages by replacing wide glasses with tall skinny ones [10], [12], [13]. When pouring frugally in relation to the capacity of the glass, that is when pouring a drink in an extremely large American glass [10], or a shot in a regular glass [11], people may serve more in a short wide than a tall skinny glass. In contrast, when generously filling up a regular-sized glass, people serve more in a tall narrow than a short wide glass. Apparently, there are common circumstances that evoke people to pour more sugary beverage in tall skinny than short wide glasses, and when people drink what they pour, this may positively impact the consumption volume in a single-serving context.

When maximizing a filling, people might use a heuristic to prevent spillage. A possible pouring strategy might be to bring the liquid level to a proper distance relative to the brim of the glass. When this reference distance is fixed and independent from glass shape, we can expect to find the glass elongation effect that was found in the present study; People would serve more in the tall narrow than the short wide glass. Yet, our data indicated that not only the poured volume, but also the level of the liquid from the brim, was significantly affected by glass shape, when the generous pourers served a drink (t(13) = 4.9, P<.001, d = 1.3). For the generous pourers, the pouring volume was less, but the liquid level that was reached was closer to the brim for the short wide (2.42 cm) than the tall narrow glass (3.04 cm). Note that the liquid may slosh more easily back and forth in a wide glass than in a narrow glass. In the light of spillage, the wide glass would therefore require to be filled even less close to the brim than the narrow glass. Thus, we conclude that reducing the risk of spillage does not fully explain why people serve more in the tall narrow than the short wide glass.

The results of experiment 1 are consistent with the perspective that elongation may influence the perceived volume of both the filled and the unfilled part of the glass and that focusing on a different part of the glass, when pouring, may result in a differential effect of glass elongation on the volume poured. To further explore this idea we performed a second experiment where we assessed the influence of task instructions that may focus attention to the unfilled or filled part of the glass.

## Experiment 2

Experiment 2 tested the hypothesis that the glass elongation effect on volume poured could be reversed by changing the focus of attention from the filled to the unfilled part of the glass. Elongation may positively influence the perceived volume poured, which might prevent people from pouring more, when focusing on the filled part of the glass. It is therefore expected, that less liquid is poured in the tall narrow glass than in the short wide glass, when the instruction is to pour a designated amount. Elongation of the free volume in a glass may positively bias the perceived capacity of the unfilled part of the glass, and correspondingly, less free space may be spared in the tall skinny glass than in the short wide glass, when focusing on the unfilled part during a pouring task. In accordance, people are expected to serve more liquid in the tall narrow glass than in the short wide glass, when the instruction is to leave space in the assigned glasses for the addition of a designated amount.

### Methods

#### Participants

24 participants (62.5% female, mean age 37.9 (±13.6) years, range 23–68) volunteered to participate in a study from the Centre of Human Movement Sciences. Written informed consent was obtained from all participants involved in the study. The local ethics committee of the Centre of Human Movement Sciences of the University Medical Centre Groningen approved the experiment.

#### Glasses and instructions

The glass shapes that were evaluated were similar to experiment 1: a tall narrow glass (height 14.5 cm, unfilled weight: 274 gr) and a short wide glass (height 7.5 cm, unfilled weight: 315 gr). Both glasses could contain 0.3 liters (10.1 fl oz). Participants were instructed to pour water from a 2.5 liters jug (filled with 1.5 liters) into a glass that was provided by the experimenter to the participant. To focus attention on the filled part of the glass participants were instructed to fill the glass with a given amount. To focus attention on the unfilled part of the glass participants were instructed to fill the glass but leave space for a given amount. The given amount of liquid was presented to the participants by showing a small reference glass, i.e. a cylindrical shot glass with a height of 5.5 cm and a capacity of 50 ml (1.5 fl oz), or a larger reference glass, i.e. a Duralex Picardie glass with a height of 7.7 cm and a capacity of 170 ml (5.7 fl oz).

#### Set-up and procedure

After signing the informed consent participants were seated at a desk. On the desk there was a 2.5 liters (84.5 fl oz) jug filled with 1.5 liters (50.7 fl oz) water. At the start of a trial the experimenter placed an experimental glass (tall or short) at the desk in front of the participant and a reference glass (small or large) at a distance from the participant, so they could glance at the reference glass while pouring. The experimenter instructed the participants either to fill the glass with an amount that equals the amount that could be contained by the reference glass or to fill the glass but to leave space for an additional amount that could be contained by the reference glass. Participants were allowed to touch the experimental glass while pouring. After a trial the filled glass was picked up from the table by the experimenter and weighed. After weighing, the glass of water was emptied in the jug. Most participants guessed that the two experimental glasses had an identical volume. In total, participants performed 8 trials that differed in instruction (intended volume or rest volume), amount (small or large), and glass shape (tall or short) that were counterbalanced to control order effects.

### Results

Table 2 presents the means and standard deviations for the volume poured in each of the eight conditions, that is for both instructions (intended volume versus rest volume), both glass types (tall versus short) and both required amounts (small versus large). A significant two way interaction of instruction×glass type (F(1,23) = 13.77, p = .001, η_p_^2^ = .37) was found, revealing the reversal of the glass elongation effect when the instruction focused attention on either the filled or the unfilled part. When the instruction was to pour an amount that equals the reference value participants poured significantly more in the short wide than in the tall narrow glass (t(23) = −2.92, p = .008, *d* = −0.59). On the contrary, when participants were instructed to leave space for an amount that equals the reference value participants poured significantly more in the tall narrow than the short wide glass (t(23) = 3.52, p = .002, d = .72). As expected given the task instructions, also a significant main effect of instruction (F(1,23) = 179.21, p<.001, η_p_^2^ = .89) and a significant interaction of instruction×amount (F(1,23) = 428.32, p<.001, η_p_^2^ = .95) were found. In both the tall and short glass participants poured a small volume when pouring the shot and a large volume when leaving space in the glass for an additional shot. When the larger reference glass was presented, no significant differences in the volume poured were found between the rest volume and intended volume conditions.

**Table 2:** The mean quantity of water poured and standard deviations (in grams), the mean absolute error of the poured volume with regard to the required volume (in grams), the mean percentage of the glass filled, the liquid level relative to the rim of the glass (in cm), percentage of participants that poured more in the short wide than in the tall narrow glass (% pp_negative bias_) as a function of Instruction (fill or leave space), Amount (small versus large) and Glass (tall narrow versus short wide).

Instruction	Amount	Glass	Quantity (g)	Absolute error (g)	% filled	% pp_negative bias_
Intended volume	small	tall	42.4 (13.5)	−7.6	13	92
		short	56.3 (18.1)	6.3	18	
	large	tall	141.1 (26.7)	−28.9	45	58
		short	148.0 (26.9)	−22.0	47	
Rest volume	small	tall	241.1 (20.8)	−23.9	77	17
		short	221.5 (27.3)	−43.5	70	
	large	tall	142.2 (30.2)	−2.8	45	33
		short	131.4 (34.1)	−13.6	42	

### Conclusion

Experiment 2 revealed a negative glass elongation bias on poured volume, when the instruction was to pour an amount that equals a reference volume (i.e. the intended volume strategy), and a positive glass elongation bias on poured volume, when the instruction was to leave space for an amount that equals a reference volume (i.e. the rest capacity strategy). The instruction to pour a given amount might have focussed attention on the filled part of the glass, and elongation of this filled part may have allured people into pouring less in the tall narrow glass than in the short wide glass. The instruction to save a certain rest volume in the glass may have directed attention to the unfilled part of the glass, and elongation of this unfilled part may have allured people into pouring more in the tall narrow than in the short wide glass.

## General Discussion

The first important finding of this study is the observation that glass elongation differentially affects the volume served when filling a glass generously instead of sparingly. Previous studies, where people served sparingly with regard to the capacity of the glass, claimed that more liquid was served in short wide than tall narrow glasses [10], [11]. In the present study, this effect was also found when people poured a small amount (a shot) in glasses that could contain 0.3 liters. In contrast with the prior studies [10], [11], a reversal of this glass shape effect on the volume served was found when the glasses were filled close to capacity. Generous pourers served more in the tall narrow glass than in the short wide glass when helping themselves to a drink of lemonade. This present finding puts the elegantly simple diet advice in perspective to reduce overconsumption of sugary beverages by replacing short wide soft drink glasses with tall slender ones.

Secondly, and more important, experiment 2 allowed us to determine that a reversal of the glass elongation bias on the volume served could be evoked by means of explicit pouring instructions that shifted attention from the filled to the unfilled part of the glass. Elongation of the poured liquid volume, at the bottom part of the glass, may result in an overestimation of the poured volume [9], which, in turn, might restrain the total amount of liquid poured [10], [11]. In line, it was observed in this study that more liquid was poured in the short wide than the tall narrow glass, when the explicit instruction was to pour an intended volume. Due to the same visual illusion, the elongated volume that is unfilled, at the top of the glass, appears larger as well. Perceiving that the elongated glass has more rest capacity available might evoke pouring more. Consistently with this notion, the glass shape effect on the volume poured was found to reverse when the explicit instruction was to leave a certain volume in the glass unfilled.

Allocating visual attention to places where information is needed for executing the pouring task is important [17]. When pouring, eye movements are necessary for locating objects, guiding the movements of the jug to the glass and also for checking when the liquid reaches the right level to stop pouring. Two different information-based pouring strategies might be distinguished, the *rest volume strategy* and the *intended volume strategy*. In order to prevent overflowing, it is likely that generous pourers control the liquid level with respect to the top of the glass when filling the glass near capacity. A similar strategy can be expected when people aim to leave a designated free space in the glass. Elongation of the free space in the glass may bias the pourer to serve more in the tall narrow than the short wide glass. In contrast, people might control the liquid level with respect to the bottom of the glass when pouring an intended quantity in a relatively large container that can hold far more. Adopting an “intended volume” strategy is plausible when pouring a designated volume, such as a shot, and when pouring sparingly in a glass with a relatively large capacity. Elongation of the filled volume in the glass may bias a pourer to serve a larger shot in a small wide than a short narrow glass. Task goals (serving an adequate amount or leaving enough space unfilled in the glass), task context (type of beverage, risk of sloshing, point of view) and individual factors (being thirsty, vigilantly monitoring food intake, clumsy in pouring, cautious or generous in serving) may all affect where people fixate their gaze to assess the nearness of the liquid level to a target level while pouring.

Previous research of Krishna [18] showed that when attention is diverted away from vision, to the haptic modality, the diameter of the glass is the most salient dimension for making volume judgements. Since, the short wide glass has the bigger diameter it is judged to have a larger volume when participants are blindfolded. When both haptic and visual input was available estimates were found to be comparative to that of vision alone. So, even though haptic contact with the glasses was permitted in the present study, it was probably not a potential confounder. Based on the findings in this study we can hypothesize that the elongation bias reversal was the result of a shift in the allocation of *visual* attention to different parts of the glass (i.e., the bottom or the top) based on task and individual factors. This needs to be further explored in future research. By introducing eye movement monitoring or visually augmented cues that may potentially direct attention to the upper or lower part of the glass while pouring a drink, we can perhaps provide more evidence for a difference in perceptual style based on task constraints.

The managerial importance of fully understanding the boundary conditions that reverse the glass elongation effect on volume poured becomes clear when looking at consumption intake. Beverages represent a substantial source of calories [19] and studies show a positive relationship between consumption of sugar-sweetened beverages and obesity in both children and adults [19], [20]. Children typically drink lemonade from elongated cups and glasses, which accommodate small hands. If we assume that these glasses are generally filled to capacity, the positive elongation bias discussed would suggest that children might be prone to overserving and correspondingly overconsuming. Given the clinical concern and global epidemic of being overweight, the research findings might initially suggest that people should be informed about this elongation bias. The reality is however that knowledge has little impact. After informing participants about the bias in glass shape, and providing practice trials, people still showed an effect of elongation on pouring volume. The elongation bias affects both novices and experts and appears to be stronger than our vigilance [11]. Based on the prior studies [10], [11] which were conducted in the U.S., where everything is bigger including the capacity of glasses, dieticians world-wide might advise us to reduce caloric intake by replacing our short wide glasses with tall slender ones. Yet, the elongation effects discussed in the present paper suggest that there are common circumstances, such as filling up a glass of a smaller size, that may invoke quite the reverse. Apart from the task context, also individual factors may play a role in changing the allocation of visual attention for pouring. People who control their food intake (for example, diabetics or people with an obsessive fear of weight gain) might focus more on the filled part of the glass to serve an intended volume, and this may evoke a negative glass elongation bias on the volume poured. Possibly, people who are clumsy in handling objects might focus more on the unfilled part of the glass to prevent it from overflowing, and this allocation of visual attention may evoke a positive elongation bias on pouring. It seems crucial to scrutinize the boundary conditions, in order to fully understand the effects of glass elongation on served drink volume. Individual factors and contextual factors can all impact the viewing strategy of the participant, and may reverse the glass elongation bias on the volume served.
